# A Physiologist's View of Smoking

**Published:** 1892-02-20

**Authors:** 


### A PHYSIOLOGIST'S VIEW OF SMOKING.

If one can accept all the hysterical and polemical nonsense which has been introduced into the newspaper warfare against the luckless woman, who occasionally solaces herself with a cigarette, one must perforce believe that moral, social, and physical degradation follows as the inevitable and remorseless Nemesis of such indulgence.

As far as the moral and social side of the question is concerned, we will leave the defence in the hands of the lady advocates themselves. On this battlefield they can easily hold their own, without extraneous or expert assistance; as "selfish man," our own view of the question has been prettily expressed by the late J. K. Stephen in the following lines to a lady who smoked :—

"Spare us the hint of slightest desecration,
Spotless preserve us an untainted shrine;
Not for thy sake, O Goddess of Creation,
Not for thy sake, O woman, but for mine."

But with regard to the third point, namely, the physical aspect of smoking, perhaps a few words from the pen of a physiologist may be received with favour by those who see no harm in smoking.

It must still be within the memory of many that in the year 1860 England was convulsed with hystero-epileptic fits of tobaccophobia. The papers devoted columns, nay, pages, to the discussion of this question, and a petition to Parliament was the inevitable sequence. As a result of the calm and dispassionate survey of the evidence, Dr. Richardson, in the light of the knowledge then at his disposal, formulated the following verdict: (1), The effects of smoking are very transitory; (2) the evils are functional in their character, and statements that it causes insanity, epilepsy, St. Vitus dance, apoplexy, organic diseases of the heart, cancer, and consumption, are devoid of truth; (3) the habit of smoking is deleterious to the young; (4) tobacco is a luxury, but, probably one of the least hurtful of all luxuries." Although an enormous amount of research work has accumulated since that date on the subiect of the physiological action of tobacco, we can still adhere very closely to Dr. Richardson's views, with the exception, perhaps, that organic changes may take place in the fundus of the eye as the result of excessive smoking, and that cancer on the lips may sometimes be ascribed as partly due to the constant irritation of the stem of a clay pipe, but both of these accidents are so rare, and so readily to be controlled by individual moderation that they hardly deserve to be quoted as arguments against the use of tobacco.

In an article which appeared in THE HOSPITAL of February 6th, the therapeutic action of nicotine was clearly and succinctly set forth, and the train of symptoms which were there ascribed to the administration of this drug were well calculated to frighten the boldest smoker. It was, however, for these very properties that a wise dispensation, as personified in the General Medical Council, eliminated this alkaloid from the pages of the "British Pharmacopœia," justly regarding it as a very unsafe and uncertain drug, being second only to prussic acid in the rapidity of its action, a single drop being sufficient to cause death in less than five minutes. One knows, however, that all, or nearly all useful drugs are poisonous when administered in excessive doses, but that is no argument for dispensing with them when they can be given in safe and beneficial doses, such as we contend to be the case when a moderate quantity of tobacco is smoked. It may be instructive therefore to enquire into the therapeutical action of nicotine when taken in small quantities, such, for instance, as when three or four pipes are smoked during the day. The writer well remembers an experiment which he saw performed many years ago at a public meeting. A small boy was seated at a table, and by means of a sphygmograph a tracing of his heart beat was recorded on a revolving surface. At the commencement of the experiment the frequency of the beat was 72 per minute. He was then presented with a cigar and told to smoke. Almost immediately the frequency of his heart grew less, and at the end of seven minutes was less than sixty; and at this interesting period of the experiment the curiosity of the onlookers were doomed to disappointment by the rapid and precipitous retreat of the young tyro. Duly impressed with what they had seen, ladies went home to hide their husbands' pipes, and smokers to smoke no more. But, of course, it is important not to lay too much stress on a single phenomenon, such as the marked slowing of the heart, especially in the cases of novices in the art of smoking; and, moreover, in itself, slowing of the heart is not detrimental to health, and it is a consummation often devoutly wished for by physicians; and except in cases where there are certain organic diseases of the heart, one may fairly say that it does no harm at all. In cases of nicotine poisoning death is always due to stoppage of respiration and not to inhibition of the heart. In the case of the mature smoker no such marked phenomena are to be seen, and the action of the drug is as follows: In the first place fumes of tobacco when taken into the mouth act as a nervous stimulant to the spinal cord, increasing thereby what may be called the activity of the organic function of the body, that is to say, our respiratory function, the peristaltic action of the intestine, and consequently our digestion. Moreover, a pipe after dinner, by quieting the tumultuous action of our heart helps us to tide over the difficulties often associated with the first half hour after a heavy meal. By a reflex action on the mucous membrane of the mouth and nose, tobacco smoke is said to dilate the arterioles of the brain, and thus enable us to play our parts in the social duties of conversation assisted by a clear and active brain. Whether this be so or no, it is quite certain that the comfortable sensation of "bien être" which steals over us as we indulge in our pipe after dinner, is to be obtained in no other way whatsoever.

The actual amount of nicotine absorbed into the blood on smoking a pipe or cigar is probably exceeding small. In the first place about fifty per cent. of the entire alkaloid contained in tobacco is destroyed by decomposition, or escapes from the bowl of the pipe; and in the second place, unless one inhales the smoke into one's lungs, nearly all the smoke and its contained alkaloid is returned into the air after being retained in the mouth but for a short while. The amount of nicotine which is actually absorbed from a fair-sized pipe is about $\frac{1}{10}$ of a grain, and in the case of a cigar rather less. But then it must be remembered that the effects of tobacco smoke, whether pleasant or otherwise, largely depends on the decomposition products of nicotine, such, for instance, as the bodies pyridine and collidine, the proportion of these substances differing according as to whether one smokes a pipe or a cigar. And to this circumstance is probably to be attributed the fact that when we have got quite used to a pipe a cigar may have a very powerful effect, and vice versa. While then we have nothing to say against the moderate use of tobacco, any excess in this direction may be followed by all the ills to which the writer in last week's HOSPITAL called attention, and more also, which we too refrain from mentioning. Death has followed on the smoking of twenty cigars, eighteen pipes of tobacco, and also on swallowing the saturated plug of an old pipe, but all these cases, it is needless to point out, come under the category of excesses.

